# DNA barcoding reveals incorrect labelling of insects sold as food in the UK

**DOI:** 10.7717/peerj.8496

**Published:** 2020-02-11

**Authors:** Stefanos Siozios, Annie Massa, Catherine L. Parr, Rudi L. Verspoor, Gregory D.D. Hurst

**Affiliations:** 1 Institute of Integrative Biology, University of Liverpool, Liverpool, United Kingdom; 2 School of Environmental Sciences, University of Liverpool, Liverpool, United Kingdom; 3 Department of Zoology & Entomology, University of Pretoria, Pretoria, South Africa; 4 School of Animal, Plant and Environmental Sciences, University of Witwatersrand, Wits, South Africa

**Keywords:** DNA barcoding, Food science, Entomophagy

## Abstract

**Background:**

Insects form an established part of the diet in many parts of the world and insect food products are emerging into the European and North American marketplaces. Consumer confidence in product is key in developing this market, and accurate labelling of content identity is an important component of this. We used DNA barcoding to assess the accuracy of insect food products sold in the UK.

**Methods:**

We purchased insects sold for human consumption from online retailers in the UK and compared the identity of the material ascertained from DNA barcoding to that stated on the product packaging. To this end, the COI sequence of mitochondrial DNA was amplified and sequenced, and compared the sequences produced to reference sequences in NCBI and the Barcode of Life Data System (BOLD).

**Results:**

The barcode identity of all insects that were farmed was consistent with the packaging label. In contrast, disparity between barcode identity and package contents was revealed in two cases of foraged material (mopane worm and winged termites). One case of very broad family-level description was also highlighted, where material described as grasshopper was identified as *Locusta migratoria* from DNA barcode.

**Conclusion:**

Overall these data indicate the need to establish tight protocols to validate product identity in this developing market. Maintaining biosafety and consumer confidence rely on accurate and consistent product labelling that provides a clear chain of information from producer to consumer.

## Introduction

Human consumption of insects (entomophagy) is a well-established phenomenon with a widespread and diverse cultural heritage. Currently, there are more than 2000 species of insects consumed around the world (Jongema, 2017; Ramos-Elorduy, 2009). Increasing attention has been directed to the potential contribution entomophagy can make to reaching worldwide food security targets (Huis, 2013). Fighting malnutrition, increasing the sustainability of livestock production, and improving dietary healthiness are all examples of ongoing and evolving research on insects as food (Berggren, Jansson & Low, 2019; Huis, 2013; Payne et al., 2016).

Markets for edible insects exist on local, national, and international scales. Commercial availability of insects as food is emerging in regions where there is little or no traditional entomophagy, for example the EU and the US (Collins, Vaskou & Kountouris, 2019). Reliable product identification is a key feature of developing consumer confidence in these emerging markets. This chain starts with the collected material, which must be correctly identified. This should be relatively simple for farmed produce, but remains challenging for field-collected material. Morphological identification generally requires expert knowledge, specialised keys and microscopy. Further challenges are posed by mimicry (one species evolving to look like another species) and stage-specific identification (for some species, morphology-based identification is only possible during certain life stages or castes). There is an imperative to surmount these challenges to establish identity for edible insects, as mislabelling of food products has serious implications for consumer confidence (e.g., horse meat found in beef product) (Barnett et al., 2016) and food safety.

Several methods have been employed to determine the contents of insects packaged for human consumption. Ulrich et al. (2017) developed MALDI-TOF analysis of protein constitution as a tool to distinguish five commonly farmed species of insects sold in the Netherlands and Germany. This approach requires the appropriate technology base, and also requires a reference data set against which to compare the species of interest. Veys & Baeten (2018) used classical morphological taxonomy to identify the contents of insects in aquaculture feed mix. This method proved effective, but requires a high level of expertise. More recently, Kim et al. (2019) developed PCR based methods for verification for selected target species, based on COI sequence. The method developed can be deployed widely, but represents a specific test for the presence of particular material for the purpose of verification, rather than a hypothesis-free investigation as to contents.

DNA barcoding allows independent analysis of the taxonomic identity, commonly using the sequence of the cytochrome oxidase I (COI) gene within the mitochondrial genome (Hebert et al., 2003). The COI gene is amplified through the polymerase chain reaction (PCR) and then sequenced. This sequence is then compared to a reference database to identify the closest species matches to the template. The technique is widely used in food science. For instance, identifying the source of material in fish fingers and other fish produce has revealed that product content description can be misleading, both in processed grocery produce and in prepared food in restaurants (Christiansen et al., 2018; Hossain et al., 2019; Huxley-Jones et al., 2012; Willette et al., 2017). Barcoding has similarly been used to test labelling accuracy of vertebrate game products (Quinto, Tinoco & Hellberg, 2016) and ground meat products (Kane & Hellberg, 2016). Notably, on each occasion highlighted, deficits in product labelling accuracy were found.

In this study, we investigated the accuracy of labelling of commercially available insect material presented for sale in the EU. We aimed to establish whether the identity of the material as stated on the packet reflected the contents within, as ascertained through DNA barcode analysis. To this end, we purchased insects for human consumption from online suppliers in the UK, amplified and sequence the COI barcode gene, and compared barcode identity to that stated on the packet.

## Methods

Preserved and prepared insects intended for human consumption were purchased from four commercial suppliers in the UK: Crunchy Critters (https://www.crunchycritters.com/), EatGrub (https://www.eatgrub.co.uk/), Your South African Shop (https://yoursouthafricanshop.co.uk/) and Zimtuckshop (http://zimtuckshop.co.uk/). Note, supply of products with a country of origin outside the EU have been discontinued since the study. Between one and ten individual insects were removed from each packet, and DNA template was prepared using the Promega Wizard Kit according to manufacturer’s instructions. COI barcode amplicons were generated by PCR using a variety of primer combinations: C1N/C1J, HCO/LCO, MLepF1/LepR1 (Folmer et al., 1994; Hajibabaei et al., 2005; Hebert et al., 2004; Simon et al., 1994) (see Table 1). DNA preparation and amplification were completed in a dedicated PCR cabinet, and the target species had not been present in the physical laboratory space previously. PCR reactions using *Drosophila melanogaster* DNA and without DNA template were served as positive and negative controls respectively.

**Table 1:** Description of insect food products supplied for human consumption and barcode identity of material. Material obtained for testing, including packet description is given and likely source (farming or wild collection), with numbers of individuals for which barcodes were obtained. Primer combination used for amplification is given for each product type, with barcode identity of the material tested ascertained from the COI sequence alongside accession number for this sequence.

**Product type**	**Farming method**	**Supplier**	**Packet description**	**Individuals tested (packets tested)**	**COI primer combination^a^**	**BOLD database**		**NCBI GenBank**	**Accession #**
						Barcode identity (genus or species level)	Probability (%)	GeneBank best hit (% ident.)	
Mopane worm,	Likely wild collected	1	Mopane worm	10 (1)	C1J/C1N	*Gonimbrasia*	100	*Gonimbrasia alopia* (92.9)	MN176162
								*Gonimbrasia alopia* (92.5)	MN176163
		2	Mopane worm, *Gonimbrasia belina*	20 (2)	C1J/C1N	*Gonimbrasia*	100	*Gonimbrasia alopia* (92.9)	MN176160
								*Gonimbrasia alopia* (92.4)	MN176161
		3	*Gonimbrasia belina*	2 (1)	C1J/C1N	*Gynanisa*	98.9	*Gynanisa maja* (95.3)	MN176159
Buffalo worm	Farmed	3	Buffalo worm, *Alphitobius diaperinus*	2 (1)	MlepF1/LepR1	*Alphitobius diaperinus*	99.7	*Alphitobius diaperinus* (99.7)	MN176146
		4	Buffalo worm	2 (1)	MlepF1/LepR1	*Alphitobius diaperinus*	99.7	*Alphitobius diaperinus* (99.7)	MN176147
Mealworm	Farmed	3	Meal worm, *Tenebrio molitor*	3 (1)	C1J/HCO	*Tenebrio molitor*	100	*Tenebrio molitor* (100)	MN176155
									MN176156
		4	Mealworm	2 (1)	C1J/HCO	*Tenebrio molitor*	100	*Tenebrio molitor* (100)	MN176157
									MN176158
Queen Leaf cutter ants	Likely semi-wild collected	3	QLC ants, *Atta laevigata*	2 (1)	C1J/C1N	*Atta laevigata*	100	*Atta laevigata* (100)	MN176153
Grasshopper	Farmed	4	Grasshopper	3 (1)	C1J/C1N	*Locusta migratoria*	100	*Locusta migratoria* (100)	MN176151
									MN176152
Locust	Farmed	3	Locusts, *Locusta migratoria*	1 (1)	C1J/C1N	*Locusta migratoria*	100	*Locusta migratoria* (100)	MN176154
Termite	Wild collected	3	Termite, *Nasutitermes costalis*	2 (1)	LCO/HCO	*Odontotermes* sp.	99.5	*Odontotermes* sp. (99.3)	MN176164
						nd	nd	*Odontotermes* sp. (95.6)	MN176165
Cricket	Farmed	3	Cricket, *Acheta domesticus*	3 (1)	LCO/C1N	*Acheta domesticus*	99.8	*Acheta domesticus* (99.7)	MN176148
						*Acheta domesticus*	100	*Acheta domesticus* (100)	MN176149
		4	Cricket	2 (1)	LCO/C1N	*Acheta domesticus*	100	*Acheta domesticus* (100)	MN176150

**Notes.**

a PCR cycling program: [95 °C for 5 min; 35 × (95 °C for 30 s, 54 °C for 15 s, 72 °C for 90 s); 72 °C for 10 min].

Amplicons were purified through an EXOSAP reaction, and then sequenced using the original primers using the Sanger method by Eurofins Genomics (Ebensburg, Germany). Amplicon Sequences were curated manually, establishing high quality (QS > 40) sequence, removing priming end sites, and where appropriate for phylogenetic analysis, creating a consensus using Geneious software v6.1.8. Similar sequences in the national centre for biotechnology information (NCBI) and the Barcode of Life Data System (BOLD) database (BOLD: Ratnasingham & Hebert, 2007) were then ascertained through BLAST searching and BOLD database barcode match. Sample identification was performed by searches against BOLD “species level” database which assigns probabilities of placement in a taxon. These results were compared with the one obtained through NCBI BLAST searches. Where there was no clear match, the most similar sequences on NCBI/BOLD were retrieved, and the phylogenetic position of the target sequence estimated relative to these, to provide broad scale information as to the taxonomic affiliation of the target specimen. Bayesian phylogenies were estimated using MrBayes v3.2.6 (Ronquist et al., 2012) by sampling across the GTR+G model space (lst parameters: nst = mixed, rates = gamma). MCMC settings were as follows: two independent runs were performed for 1,100,000 generations and sub-sampling every 200 generations using four Markov chains. The first 100,000 samples were discarded as burn-in.

## Results

Purchased material varied in the precision of contents labelling on the packet. In terms of content identity, some manufacturers specified the contents to Latin binomial species name, others by the common name (e.g., ‘mopane worm’), and others characterised products more generically (e.g., ‘grasshopper’ ‘cricket’). Country of origin was noted by all suppliers, and whether the material was farmed or field collected noted by three of four suppliers. Allergy advice was supplied on material from two of the four suppliers. For one supplier, this was in the form of a precise allergen advisory emphasising consumers allergic to shellfish may also be allergic to insects. For another supplier, it was indicated less precisely that the products were Crustaceans in terms of allergen profile. Detailed nutritional information was provided by one of the four suppliers.

Valid COI barcodes were obtained for 53 individuals from 14 packets representing 8 product types, with some product types offered by multiple suppliers (Table 1, Genbank accessions MN176146 –MN176165). There were 12 packets where the DNA barcode of all individuals tested corresponded to that stated on the packet. However, there were two cases where the barcode identity was at variance with that stated on the packet.

**Figure 1:**
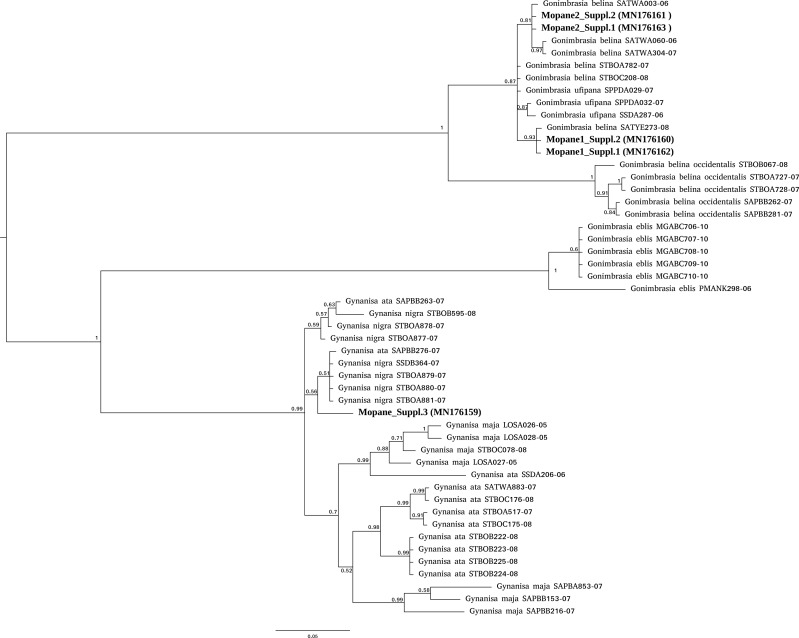
Phylogenetic affiliation of COI barcodes for specimens marketed as mopane worms. COI barcodes were generated for material from three suppliers. The affiliation of these compared to *Gonimbrasia belina* was estimated using MrBayes v3.2.6 using MCMC under a GTR + G model. Posterior probabilities are marked on each node.

For mopane worms, barcode identity was consistent with the packet (*Gonimbrasia belina*) for three packets sourced from two suppliers but mismatched for a third supplier. For this third supplier, the barcodes obtained (*N* = 2) did not have a strong match in either BOLD or NCBI databases and nucleotide divergence from *Gonimbrasia belina* reference sequences in BOLD database was between 11–13% . Phylogenetic analysis placed these specimens as an unknown saturniid moth in the genus *Gynanisa* (Fig. 1). For the three packets where barcode was consistent with packet identity, the barcodes fell securely in the genus *Gonimbrasia*, but the precise species affiliation is uncertain, as *G. belina* itself is very diverse on the database, and sequences assigned to other *Gonimbrasia* species as ingroups within these. Thus, barcoding here cannot absolutely verify the tested specimens are *G. belina*, but the data is consistent with this.

**Figure 2:**
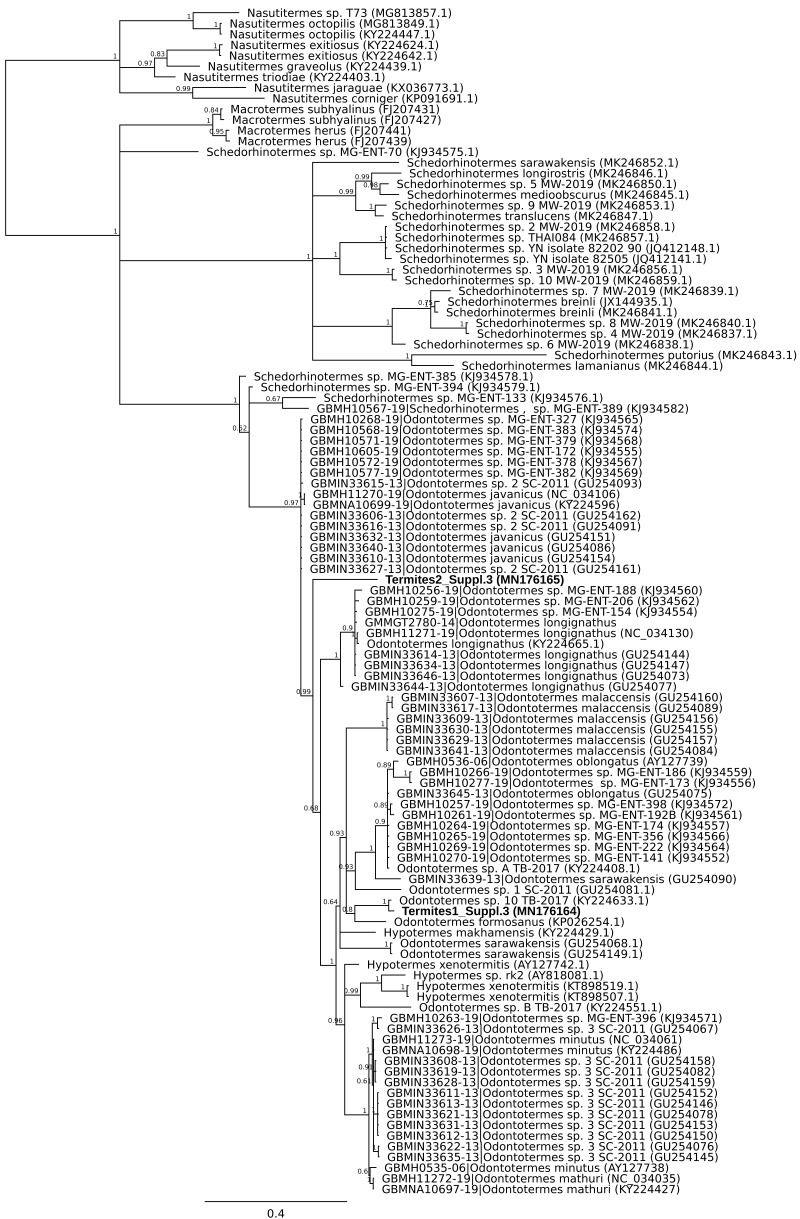
Phylogenetic affiliation of COI barcode for two specimens marketed as *Nasutitermes costalis*. A COI barcode was obtained and the relationship of this to other termite barcodes estimated using MrBayes v3.2.6 using MCMC under a GTR + G model. Posterior probabilities are marked on each node.

A second mismatch between product description and barcode identity was from the packet labelled as flying termites, *Nasutitermes costalis*, with country of origin specified as Thailand. Two distinct DNA barcodes were obtained from specimens from this packet. One matched *Odontotermes*, a distantly related genus, the other *Schedorhinotermes* sp., but neither fell into an established barcode bin (Fig. 2). The barcode IDs are compatible with the country of origin, whereas *N. costalis* is a New World species.

DNA barcodes aided in the identification of material that was broadly labelled. Material from one packet labelled as ‘grasshopper’ were a strong database match for *Locusta migratoria*. This species may be viewed as a grasshopper in the broad sense but would be more correctly labelled as either a locust or by using the precise Latin binomial.

## Discussion

Europe represents an emerging market for insects as part of the human diet, with a variety of species available for purchase from internet-based suppliers and mainstream high street outlets. The rising use of insects in the European grocery basket reflects in part a desire for more sustainable animal protein with fewer ethical concerns. Alongside this, there is also a curiosity-driven market interested in novel foods. Developing this market chain will depend in part on consumer confidence in product, which reflects both the product being as described, and being reliably safe. These elements of course work together—being accurately described is a component of being reliably safe. Further, insects for human consumption must be approved through EU novel foodstuff regulations, and material for sale must correspond to the species listed in the novel foods annex. There is also an additional value in correctly identifying wild harvested insects in developing sustainable management, licensed harvesting, and compliance with CITES and import requirements.

Product labelling of ‘farm produced’ material (crickets, grasshopper, locusts, mealworms, buffalo worms) was accurate and this likely reflects the greater security gained from the farming practice and supply chain. Our testing has limits—we dipstick tested one to three individuals per supplier in each case—but within these limits we found no cause for concern. However, in some cases product labelling was broad scale, referring to very generic groups that cover a very broad range of biodiversity. For instance, the barcode identity for a product labelled grasshopper was *Locusta migratoria*. Given the products contain a single species from farms, and not a mix, we would recommend the packaging reflects this by additionally relating the precise designation in the detailed ingredients list. This measure would make labelling consistent with other animal products, where species designations—either common or Latin—are presented, but still permits marketing using common names that consumers relate to and are attracted by.

Product labelling for foraged material was less secure. Our results showed two cases where there was a discrepancy between the product description and our barcode identification of the material. For mopane worm, which is both farmed and wild-harvested in a number of countries in Africa, barcode identity matched packet description for two of three suppliers, but was not a match for a third. In this case, whilst the material supplied was broadly of the correct group (saturniid moth), barcode identity was to a different genus of saturniid moth that inhabits the same region. For the case of the termite, the evolutionary distance between product description and our barcodes is considerable (estimated last common ancestor ∼45–75 million years ago: Bourguignon et al., 2016). The barcode species were compatible with the stated country of origin, whereas the termite named on the packet is from a different biogeographic zone. Consistent with this material being winged termites, material fell into two distinct barcode bins, which reflects the difficulty in obtaining pure samples in an environment with multiple species undergoing coordinated emergences.

Wild harvested material will present a particular challenge for accurate labelling, as there will likely often be a discrepancy between the training level of collectors and the level required for accurate species identification. We would recommend any wild material is regularly ‘dipstick’ tested for identity to ensure high standards of product identity. The COI barcode method is a possible means for doing this, but this process is in reality both laborious and too expensive for extensive application. Target-specific PCR assays, for instance based on ITS regions, should be developed to test identity more simply on a wider scale, with assays developed for species as they enter the marketplace (Kim et al., 2019). These methods would also be useful in verification in import/export as these markets develop.

## Conclusion

Our data indicate that the edible insect market presents similar challenges to others in terms of product labelling accuracy. These problems are most acute for foraged material, where identification skills are key in accurate product description. Implementation of quality control checks on product identity will be important in building the market for insects as food in Europe, as markets as a whole suffer from local failures.

##  Supplemental Information

10.7717/peerj.8496/supp-1 Supplemental Information 1 COI barcode sequence for insect material

## Additional Information and Declarations

### Data Availability

The following information was supplied regarding data availability: The COI barcode sequences are available at Genbank: MN176146 to MN176163.
